# From microbial membrane proteins to the mysteries of emotion

**DOI:** 10.1016/j.cell.2021.08.018

**Published:** 2021-09-24

**Authors:** Karl Deisseroth

**Affiliations:** 1 Department of Bioengineering, Stanford University, Stanford, CA 94305, USA; 2 Department of Psychiatry and Behavioral Sciences, Stanford University, Stanford, CA 94305, USA; 3 Howard Hughes Medical Institute, Stanford, CA 94305, USA

## Abstract

On the occasion of the 2021 Lasker Basic Medical Research Award to Karl Deisseroth, Peter Hegemann, and Dieter Oesterhelt (for “the discovery of light-sensitive microbial proteins that can activate or deactivate individual brain cells—leading to the development of optogenetics and revolutionizing neuroscience”), Deisseroth reflects on this international collaboration, his basic mechanistic and structural discoveries regarding microbial channels that transduce photons into ion current, the causal exploration of brain cell function, and the pressing mysteries of psychiatry.

Like many of my fellow psychiatrists, with each new clinical encounter, I am dismayed and enthralled alike by the profundity of the challenge facing us. No other field of medicine lacks, so fundamentally, physical interpretations of the disabling symptoms. What is the material nature of anhedonia in depression, or of debilitating anxiety, or of dissociation in response to trauma, in the same sense that congestive heart failure can be physically understood as the weakening of a pump? Due to our limited physical understanding, psychiatric disorders continue to cause worldwide morbidity and mortality historically comparable to, or greater than, that of any other class of illness. And while the COVID-19 pandemic reshapes epidemiology, this changing landscape has brought even greater urgency to our search for understanding in psychiatry. Trauma and stress experienced across the globe have intensified symptom expression in mental illness, and most of my new patients these days frame the acute exacerbations of their suffering in terms of the pandemic and its consequences.

This hope for physical understanding not only brought me to the field of psychiatry but also sustained me from my years of residency training through the past 20 years of caring for patients with mental illness. This aspiration also guided my laboratory to develop an approach for mapping individual features of adaptive and maladaptive brain states onto causal and precisely defined elements of neural circuitry, for which the framework emerged not from clinical medicine but from fundamental biochemistry, with our detailed structural and mechanistic studies of membrane proteins from single-celled algae.

My earliest formal education in biochemistry (with the Harvard crystallographers Don Wiley and Steve Harrison) had been a vital source of insight into the unique capability of high-resolution mechanistic studies to reveal deep mysteries of biology. As it turned out, my group’s elucidation of the precise roles and positioning of individual atoms in microbial light-activated membrane proteins allowed us to explore the operation of biological systems as large and complex as the mammalian brain. In so doing, we illuminated the cellular-activity basis for survival drives as fundamental as thirst and hunger, the intricate cellular dynamics involved in sensation and perception, and even complex cognitive processes such as social interaction—all by operating at the elemental level of individual atoms and cells.

Coming full circle over the past 20 years, we now can approach causal and material understanding of psychiatric disease-related states (reviewed in Deisseroth, 2015) such as anxiety and dissociation, at the level of highly resolved physical components (e.g., Kim et al., 2013). Top-down and bottom-up approaches to science have met in the middle, in the discoveries recognized by the Lasker Foundation in 2021. This story exemplifies the power of taking a molecular approach to explore complex biological systems—even for challenges as mysterious as brain function, the nature of emotions, and the disorders that define psychiatry. The hope brought to psychiatry by detailed studies of microbial membrane proteins is, I think, an uplifting story for all to share.

The story has its deepest roots in botany from more than 150 years ago, in Andrei Faminzin’s study of phototaxis in *Chlamydomonas* algae, exploring a plant behavior now known to be mediated by channelrhodopsins (Deisseroth and Hegemann, 2017). A century later, independent scientific threads would converge with Faminzin’s discovery, beginning with the biochemical identification in archaea of the broader family of microbial light-activated proteins by Dieter Oesterhelt and colleagues (Oesterhelt and Stoeckenius 1971). The first-identified member of this family of microbial proteins was bacteriorhodopsin, a light-activated ion pump (which, as a rhodopsin, uses its covalently bound retinal chromophore molecule to absorb light—and, as a pump, can transport one ion across the membrane for each absorbed photon).

This discovery was followed more than two decades later by identification of the channelrhodopsin subfamily in *Chlamydomonas* (these proteins form transmembrane channels, allowing many ions per photon to pass) as shown in work led by Peter Hegemann (Harz and Hegemann, 1991; Nagel et al., 2002). Then, over nearly another two decades, we were able to resolve the inner workings of channelrhodopsins by combining our high-resolution structures of the three major classes of these light-activated ion channels (Kato et al., 2012, 2018; Kim et al., 2018; Kishi et al., 2021) with detailed mechanistic and dynamical exploration (Deisseroth and Hegemann, 2017). Finally, using new channelrhodopsins that we had discovered and created, we achieved behaviorally potent, specific control over individual cells and even multiple-cell ensembles *in vivo* (Prakash et al., 2012; Jennings et al., 2019; Marshel et al., 2019). This control was at the speed of sensation, cognition, and action—and thereby suitable for exploring the operation, and the mystery, and the failures, of the living brain.

Turning our basic science—that of microbial light-responsive proteins—into a technology for optical control of individual cells within behaving animals involved global collaboration and convergence across widely disparate fields of human endeavor. In the past, I have had opportunities to broadly review the numerous neuroscience discoveries and the many outstanding neuroscientists involved (Deisseroth, 2015), but here I offer an expanded perspective on two of my brilliant friends and colleagues from outside neuroscience: my co-winners of the 2021 Albert Lasker Award for Basic Medical Science, Dieter Oesterhelt and Peter Hegemann.

The header image shows the human side of our international cooperation; Peter and I continue to collaborate between northern Europe and the San Francisco Bay Area, but connections run even deeper that link these two regions of the globe in the study of microbial opsins. The image at lower left, of Peter and myself in 2013, memorializes a key juncture in our ~15 years of deep friendship and collaboration; we had just been drenched by a sudden cloudburst and were sheltering in a tiny restaurant after speaking at a Cambridge (UK) symposium on the 50^th^ Anniversary of the Hodgkin/Huxley/Eccles Nobel for electrical mechanisms of action potential generation. Peter and I had recently achieved red-light-driven optical firing of action potentials by creating (with our students) the C1V1 family of channelrhodopsins in 2011; this red-light capability (derived largely from the VChR1 channelrhodopsin that we discovered in 2008) had enabled optogenetic control of single cells in living mammals using two-photon illumination, reported 1 year before the photo was taken (Prakash et al., 2012). One year after the photo was taken, in 2014, Peter and I would describe transformation of channelrhodopsins from cation-conduction to anion-conduction, demonstrating hard-won insight into the light-gated channel pore (reviewed in Deisseroth and Hegemann, 2017). Both advances were critical for later neuroscience discoveries; for example, the VChR1-derived opsins would enable our elicitation of specific mammalian behaviors via ensembles of individually specified brain cells (Jennings et al., 2019), and our designed anion-conducting channelrhodopsins would enable causal identification of specific cells underlying the fundamental mammalian survival drive of thirst, described in more detail below (Allen et al., 2017, 2019).

The picture at bottom right is of Dieter Oesterhelt in spring 1970, with his son Patrick. Dieter was tenured at the time, at the University of Munich, but on sabbatical in California with a travel award from the German Science Foundation to gain electron microscopy experience in the lab of Walter Stoeckenius at UCSF. The photo (taken about the time of his discovery of bacteriorhodopsin) is set on a Pacific beach a short walk from Golden Gate Park; the background hills are the first slopes of a rising coastal range that extends south to Palo Alto and beyond, framing Stanford’s campus between the mountains and the San Francisco Bay. I love this picture both for illustrating deep human warmth set against the chill of the San Francisco coastline and for showing a scientist visiting the US from Europe at about the same time as his transformative discovery; Dieter identified the first microbial rhodopsin that year. It had been known that one method for lysing halobacteria gave rise to a subcellular fraction called the “purple membrane”; while he was performing a routine lipid extraction of the purple membrane for a colleague as a control experiment (not his official project nor his reason for being in the Bay Area), Dieter discovered that addition of an organic solvent turned the purple membrane yellow. This color shift was remarkable because it was caused by only a simple solvent change, and Dieter realized this could be due to the presence of retinal (Oesterhelt and Stoeckenius, 1971). He then showed that this purple membrane contains only a single protein, which indeed also contains retinal linked to the protein, and that this single protein functions as a light-driven proton pump—thus marking the astonishing discovery of the microbial rhodopsins 50 years ago.

Several groups then sequenced bacteriorhodopsin by degradation and purification of the fragments and, along with Dieter, studied mutants of key amino acids. The growing field’s knowledge of the inner mechanics of bacteriorhodopsin would later help Peter and I to elucidate and redesign key features of channelrhodopsins, including the retinal binding pocket that guides color tuning, the molecular gates that help determine kinetics, and the channel pore lining that sets ion selectivity (reviewed in Deisseroth and Hegemann, 2017). One particular goal of mine, in carrying out this redesign work, was to achieve the optogenetic control of single cells during behavior. While until 2008 the known channelrhodopsins were activated by blue light, I expected that light used to activate a red-responsive channelrhodopsin would scatter less in mammalian brain tissue and might allow resolution needed for individual-cell optogenetics.

After our 2008 discovery (with Peter) of the initial red light-activated channelrhodopsin (which we called VChR1), we followed up by tuning the amino-acid homologous to bacteriorhodopsin’s aspartate-85 (D85, studied by Dieter and others) in our VChR1-derived channelrhodopsins (Deisseroth and Hegemann 2017). We found that modification here (to threonine, creating a protein called C1V1_T_) indeed enabled the initial individual-cell *in vivo* work, in part because of robust responsivity to two-photon illumination via pulsed infrared lasers, which allowed targeting single cells even in light-scattering mammalian brain tissue (Prakash et al., 2012). A similar mutation we identified at this same residue (to serine instead of threonine in another VChR1-derived channelrhodopsin) indeed enabled the initial control of mammalian behavior through groups of individually specified brain cells (Jennings et al., 2019). We later discovered new channelrhodopsins even more exceptionally well-suited to single-cell optogenetics, such as ChRmine (the pump-like channelrhodopsin), which enabled brain region-wide control of individual-cell ensembles in behaving mammals when applied together with our development of advanced forms of wide-field cellular-resolution 3D holographic optics (Marshel et al., 2019). The value of the molecular and biochemical scientific tradition, where my own scientific journey had begun, was transformative for optogenetics: enabling our high-resolution structures, our cracking of the molecular mystery of the light-gated pore, and our creation of channelrhodopsins with new kinds of function, including (for neuroscience exploration) accessing individual cells in behaving animals.

It is interesting to note here that Francis Crick had long argued that another domain of the broad molecular-biological tradition—that of targeting gain- and loss-of-function interventions to elemental components of a biological system—was needed in neuroscience; in 1999 he even published his hope for a means to use light as a means for targeting activity (increased or decreased) to genetically defined cell types. Though admitting such a use of light would be “rather farfetched,” Crick realized that if implemented, such molecularly driven gain- and loss-of-function capability would reshape the landscape of the field. The power of causal investigation applies across the life sciences, even for systems like genomes and developing organisms characterized by nonlinear and overlapping pathways, with amplifying cascades and dynamic feedback. For hypothesis-testing, biologists are trained to deliver gain- and loss-of-function interventions as two mutually informative counterpoints, to validate targeting of these direct interventions, to test comparable controls (different from the hypothesized mechanistically important targets) in parallel experimental arms, to probe the dependence and effect-conditionality of one intervention relative to another, and to design the timing and magnitude of the interventions (as much as possible) to be biologically relevant. Not all of these aspirations typically are met at the same time in successful experiments across the life sciences. For example, with elegant genetic interventions—which historically have built so much of our foundational understanding of the natural world through expression of constitutively active or dominant-negative proteins, RNAi/shRNA interventions, gene knockout/transgene expression, and genome editing—the timing and magnitude of the intervention often does not compare perfectly to naturally occurring processes; optogenetics is more flexible in this regard. But if the initial direct targeting is precise, scientific discovery is nevertheless robust even in highly nonlinear living systems with complex feedback and dynamics—just as with optogenetics in the nervous system.

One of countless paradigmatic examples comes from the developmental biology of antennae in *Drosophila*, and another from the recent optogenetic study of thirst in mammals. Insect antennae are essentially evolutionarily modified legs, and gain- or loss-of-function in the *Antennapedia* gene elicits gain- or loss-of-function in antenna-versus-leg pattern-formation decisions. *Antennapedia* interventions (targeted to a single gene) give rise to complex downstream patterns of gene expression that govern the final output of body plan (much as downstream neural activity in optogenetics gives rise to specific brain states of sensation, cognition, and action), which is the goal of the initial precise intervention; here as in optogenetics, the arrow of causality that is so informative across fields lies with the initial direct targeted intervention. Not all the processes underlying leg and antenna formation are understood, but thanks in part to this kind of causal hypothesis testing, developmental biology as a field now has a functional framework for the natural logic of body-plan generation: system-wide insight, derived substantially from molecular gain- or loss-of-function work.

Indeed, we found this experimental principle to be valuable for optogenetically exploring the mammalian survival drive of thirst (Allen et al., 2017, 2019), also with gain- or loss-of-function experiments. The loss-of-function experiments (with our designed anion-conducting channelrhodopsins) were described above; gain-of-function optogenetic control (with light delivered deep in the brain via our fiberoptic neural interface approach) was found to elicit naturalistic brainwide single-cell-resolution internal representations of the basic survival drive of thirst, and drinking water when thirsty, measured by electrophysiological recording of action potentials in behaving mammals (Allen et al., 2019). As with other fields including developmental biology, and for neuroscience as Crick had hoped, the causal modulation of correctly targeted brain-circuit components elicits naturalistic states of organismal brain function and behavior, providing a molecular framework for insight into complex system-wide processes and mechanisms.

The scope of this 50-year story exemplifies how science proceeds in unpredictable ways: here, from a solvent-induced color change noticed while prepping lysed archaea, to the study of light responses in single-celled green algae, to resolving the structural mechanisms of microbial light-gated ion channels, and finally to exploration of sensory-percept discrimination in behaving mammals achieved by recruiting individually specified cells with light in order to elicit and study complex internal representations and behaviors. The story extends even to understanding physical assembly of brain states, and symptoms such as anxiety in health and disease, at the level of individual and elemental cellular components (e.g., Kim et al., 2013; reviewed in Deisseroth, 2015).

In these difficult times, there is hope for a future that is grounded in internationally collaborative and interdisciplinary science. I am deeply grateful to my lab members and collaborators over the years, to the global scientific community, and to all who help to create and preserve the spirit of worldwide cooperation that made these discoveries possible. The progress of science truly brings the many faces of humanity together.

## Figures and Tables

**Figure:**
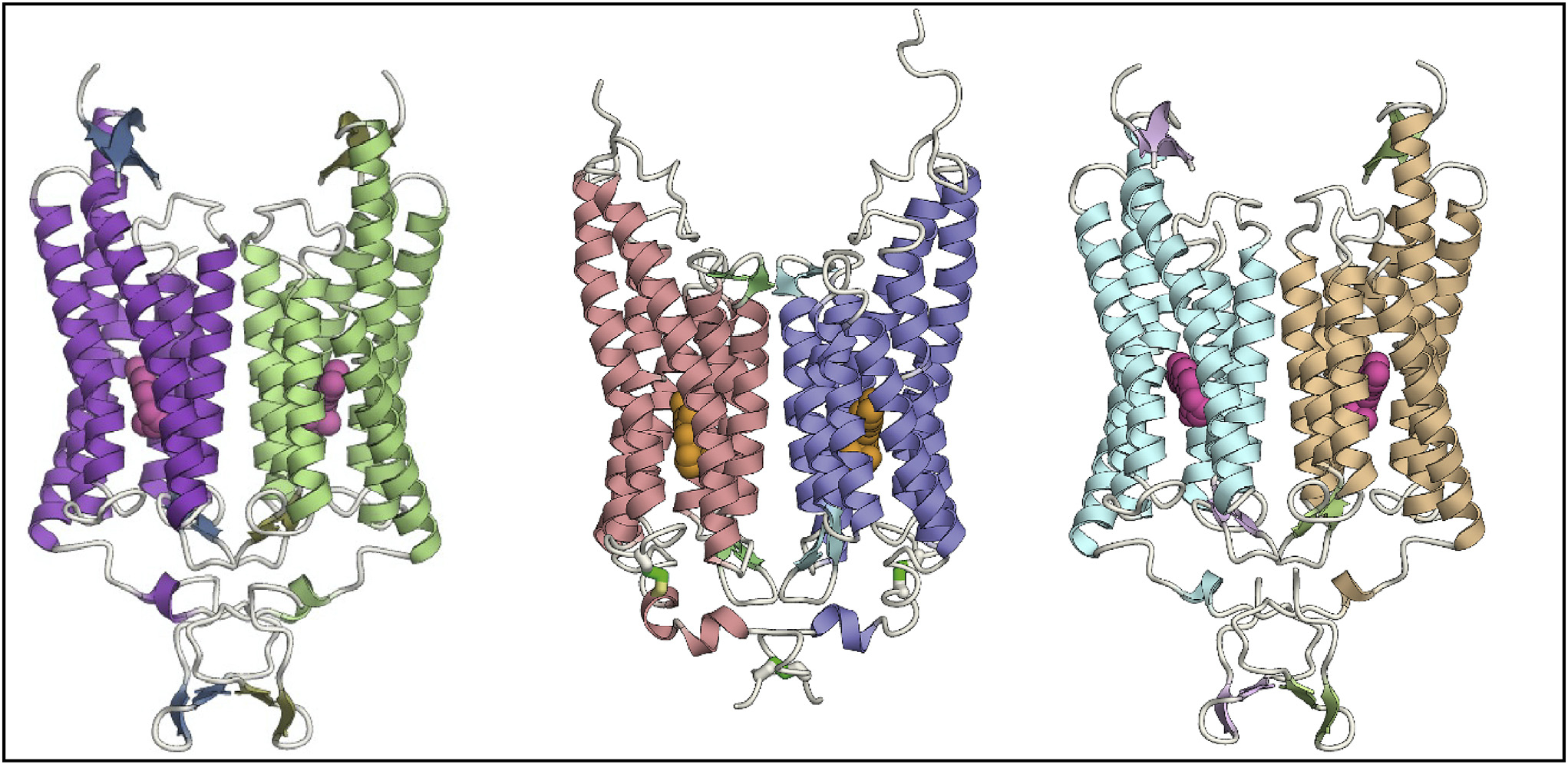
The initial high-resolution structures of the major classes of channelrhodopsins; all images are from our channelrhodopsin structure team. *Left*, naturally occurring cation-conducting channelrhodopsin (Kato et al., 2012). *Middle*/*right*, naturally occurring (Kim et al., 2018) and designed (Kato et al., 2018) anion-conducting channelrhodopsins, respectively. Note the dimeric assembly shown. Each seven-transmembrane-helix monomer displays an intrinsic pore passing near the retinal-binding pocket (all-*trans* retinal, the chromophore that absorbs the photon and triggers pore opening, is shown as the small molecule embedded within the center of each monomeric bundle of transmembrane alpha-helices). In contrast, the pump-like channelrhodopsin (ChRmine) exhibits trimeric assembly (also resolved by our channelrhodopsin structure team; see Kishi et al., 2021) and numerous structurally distinct features that may enable ChRmine (representing the third of the three major branches of the channelrhodopsin family) to function as an especially potent ion channel.

**Figure:**
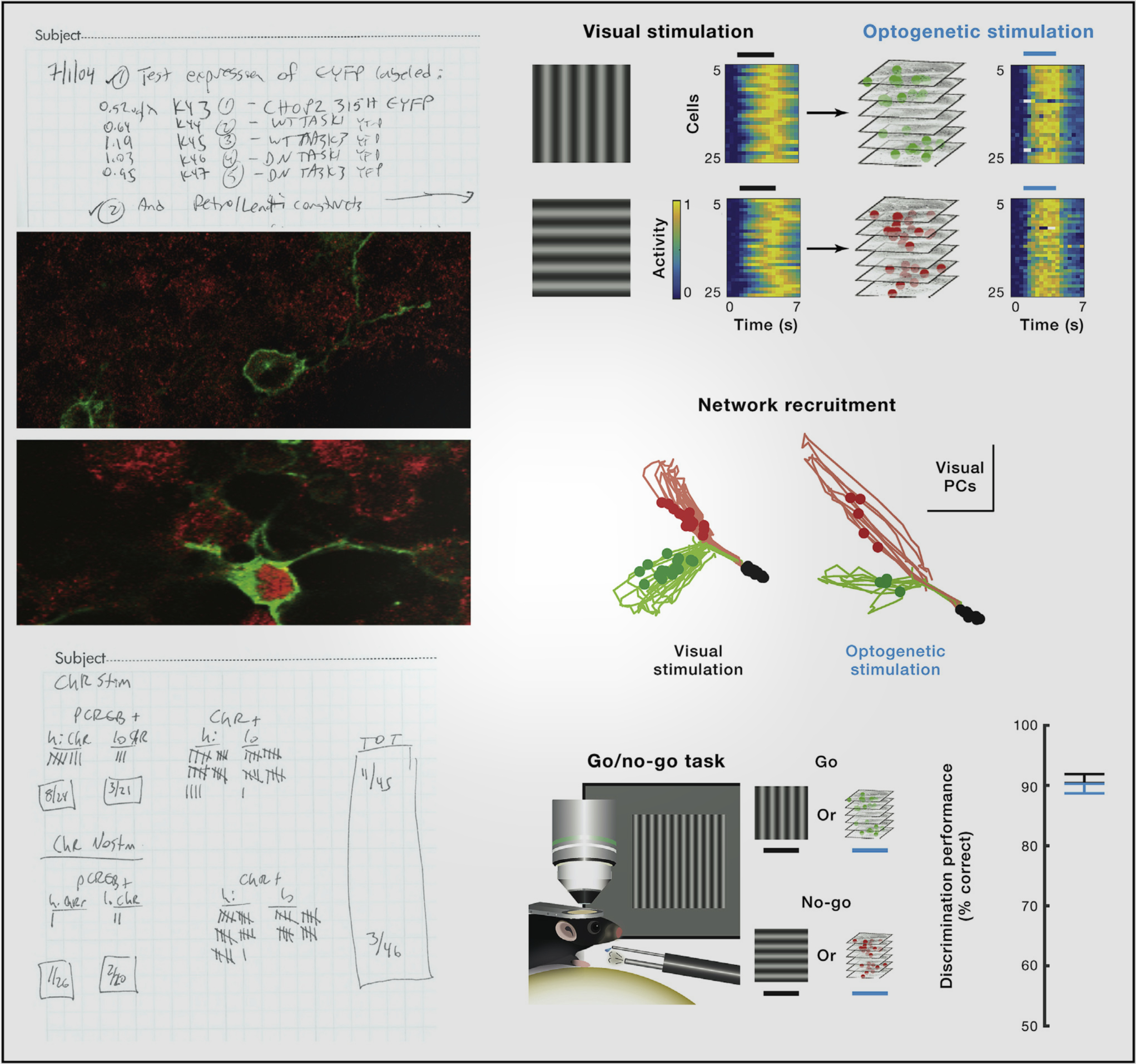
Progress of the science: microbial opsin optogenetics over 15 years (2004–2019). *Left*, my July 2004 notebook page recording the simple and humble first microbial opsin experiment in my lab (Deisseroth 2015). Testing channel families in parallel for neuronal control: constructs and plasmid concentrations that I used to transduce neurons (*top left*) are shown, including a channelrhodopsin and wild-type (WT) or dominant-negative (DN) TASK1/TASK3 K^+^ channels. *Center left*, neural expression/localization (green): subcellular distribution of fluorescent protein fused to channelrhodopsin in the ~10-μm-diameter neuronal somata and dendrites. I carried out the illumination for stimulation and the CREB Ser-133 phosphorylation (red) assay for reporting membrane depolarization 2 weeks later, and recorded cell-by-cell activation as a tally (*bottom left*; Z test, χ^2^=9.0634, d.f.=3; *P*=0.028). *Right*, optogenetic elicitation of specific discrimination behavior via defined cellular ensembles 15 years later in 2019 (*top*); cells in mouse visual cortex were selected for control with a channelrhodopsin by virtue of naturally occurring preference for responding to vertical (versus horizontal) visual stimuli. Small populations of optogenetically recruited cells (~20 or fewer) were as effective as visual stimuli in recruiting both specific behaviors (*bottom*; sensory discrimination by licking to “go” cue but not “no-go” cue) and naturalistic internal representations of the visual sensation (*middle*) tracked as recruitment of large populations of downstream cells and shown as trajectories of neural population dynamics, with dimensionality reduced to principal components (PCs; optogenetic or visual stimulus conditions shown as green for vertical, and red for horizontal; Marshel et al., 2019).
